# Antimicrobial resistance of *Escherichia coli* isolated from retail foods in northern Xinjiang, China

**DOI:** 10.1002/fsn3.1491

**Published:** 2020-03-21

**Authors:** Yingjiao Li, Mei Zhang, Juan Luo, Jiluan Chen, Qingling Wang, Shiling Lu, Hua Ji

**Affiliations:** ^1^ School of Food Science and Technology Shihezi University Shihezi China

**Keywords:** *Escherichia coli*, multidrug resistance, resistance gene, virulence gene

## Abstract

To determine antimicrobial resistance, 431 samples of retail foods purchased at different supermarkets in Northern Xinjiang were examined in this study. There were 112 *Escherichia coli* strains that were isolated, with approximately 26% of the samples contaminated by *E. coli*. The detection rate of *E. coli* isolated from pork was the highest (59.6%), followed by mutton (52.6%), retail fresh milk (52.4%), duck (36.4%), beef (35.3%), chicken (33.3%), and ready‐to‐eat food (12.9%); the *E. coli* detection rate for fish and vegetables was <11%. The result showed that the 112 isolates were mostly resistant to tetracycline (52%), followed by ampicillin (42%), compound trimethoprim/sulfamethoxazole (37%), amoxicillin (33%), and nalidixic acid (32%), imipenem resistance was not detected. One hundred isolates carried at least one antimicrobial resistance gene. The detection rate of resistance genes of our study was as follows: *tet*A (38%), *tet*B (27%), *bla*_OXA_ (40%), *bla*_TEM_ (20%), *flo*R (20%), *sul*1 (16%), *sul*2 (27%), *aad*_Ala_ (19%), *aad*B (11%), *str*A (28%), and *str*B (24%); *tet*C and *bla*_PSE_ were not detected. Virulence genes *fim*C, *agg*, *stx*2, *fim*A, *fyu*A, *pap*A, *stx*1, and *eae*A were found in 52, 34, 21, 19, 6, 3, 2, and 2 isolates, respectively; *pap*C was not detected. There was a statistically significant association between *fim*C and resistance to ciprofloxacin (*p = *.001), gentamicin (*p = *.001), amikacin (*p = *.001), levofloxacin (*p = *.001), and streptomycin (*p = *.001); between *fim*A and resistance to tetracycline (*p = *.001), ampicillin (*p = *.001), compound trimethoprim/sulfamethoxazole (*p = *.001), and amoxicillin (*p = *.003); between *agg *and resistance to gentamicin (*p = *.001), tetracycline (*p = *.001), ciprofloxacin (*p = *.017), and levofloxacin (*p = *.001); and between *stx*2 and resistance to ampicillin (*p = *.001), tetracycline (*p = *.001), compound trimethoprim/sulfamethoxazole (*p = *.002), and amoxicillin (*p = *.015).

## Graphical abstract

The drug resistance and virulence genes of *Escherichia coli* isolated from retail food in northern Xinjiang, China, were studied, and the relationship between them was analyzed to reveal the resistance of foodborne *E. coli* to veterinary and clinical commonly used drugs in the region. The paper provided reference materials to guide the rational use of antibiotics in clinical and animal feeding and control the spread of drug‐resistant strains in nature.

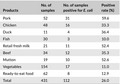

## 1 INTRODUCTION

It is well known that *Escherichia coli *mainly exists in the human and animal gastrointestinal tract. It also occurs in the natural environment, especially in soil, water, and plants (Katarzyna & Anna, 2016). Therefore, it is not surprising that some of the *E. coli* in the environment reinfects humans through vegetable‐ or animal‐derived foods.

*Escherichia coli *is a highly diverse virulent species that is widely distributed in open systems, is easy to spread in the environment, and can be harmful to human health (Tenaillon, Skurnik, Picard, & Denamur, 2010). Drug resistance genes carried by *E. coli* can be transferred to other pathogenic bacteria, and, due to the excessive use of antibiotics, selection pressure is very high, resulting in bacterial strains resistant to a variety of drugs. Multi‐drug‐resistant strains are characterized by the presence of multiple genes conferring drug resistance, which results in insensitivity to many different drug groups (Hu, Yang, & Li, 2016; Rasheed, Thajuddin, Ahamed, Teklemariam, & Jamil, 2014).

Genetic mutations or genetic acquisition of antibiotic resistance genes (ARG) through horizontal gene transfer might also result in the occurrence of antibiotic‐resistant bacteria (ARB) throughout the environment (Céline & David, 2015). This has resulted in the emergence of many different ARG, including the *dfr *and *sul* genes related to trimethoprim and sulfamethoxazole resistance, respectively (Chang, Lin, Chang, & Lu, 2007; Ho, Wang, Chow, & Que, 2009), and other genes, such as *amp*C, *oxa*2, and *tet*A.

The ever‐increasing threat of ARB may be associated with enhanced virulence (Guillard, Pons, Roux, Pier, & Skurnik, 2016; Roux et al., 2015), and with the increase in antibiotic resistance, an increase in virulence may naturally evolve. Therefore, when controlling the spread of antibiotic resistance, we must also control the spread of virulence (Meredith, Brooks, & Brooks, 2017). Although the profile of virulence and antimicrobial resistance genes of *E. coli* from foods has been reported (Luo, Ji, & Wang, 2016), the data elucidating the association between these two gene sets are lacking.

In Xinjiang, China, a previous study conducted antibiotic resistance research on foodborne *E. coli *based on samples from slaughterhouses, butcher shops, and farms (Xia, Xiang, & Guo, 2014; Yao, Long, Kuerbannaimu, Wang, & Xia, 2017). However, little is known about the resistance of those bacteria in retail foods.

There have been some reports describing the antimicrobial resistance and virulence of *E. coli*, such as Arisoy, Rad, Akin, and Akar (2008), who showed that the virulence genes *afaI*, *pap*, *hly*, *aer*, and *sfa* were increased in sensitive strains. However, detailed information on the relationship between antimicrobial resistance genes and virulence genes of *E. coli *isolated from retail foods in Xinjiang is scarce.

The purpose of this study was to evaluate the drug resistance of *E. coli* strains isolated from retail foods in northern Xinjiang, identify their virulence genes, and determine the possible relationship between the virulence genes and drug resistance.

## 2 MATERIALS AND METHODS

### 2.1 Sampling and *E. coli *isolation

A total of 431 food samples were purchased at supermarkets in Shihezi, Kuitun, and Urumqi, in northern Xinjiang, China, from 2014 to 2016, and each type of sample and its number are listed in Table 1. Each sample weighed 25 g and was placed in a sterile plastic bag containing 225 ml of sterilized sodium chloride solution (0.85%) and then homogenized for 90 s using a BagMixer 400 CC beating homogenizer. Lauryl Sulfate Tryptose (LST) broth was inoculated with 1 ml of homogenate and incubated for 48 hr at 37 ± 1°C. Gas‐positive tubes were inoculated into 100 ml of *E. coli* (EC) broth and incubated at 44 ± 0.5°C for 48 hr (Wang, Sun, & Ji, 2014). After that, one loopful from each gas‐positive tube was streaked onto eosin methylene blue agar. Presumptive *E. coli *colonies were streaked onto Luria–Bertani nutrient agar and incubated for 12–48 hr at 36 ± 1°C. Each culture was confirmed as *E. coli* through an IMViC test. *E. coli* ATCC 25922 was used as a positive control for polymerase chain reaction (PCR) of *Uid*A. Template was prepared via the boiling method, for the amplification of selected *UidA* genes in *E. coli* using PCR (Heijnen & Medema, 2006). The oligonucleotide sequences used and the predicted sizes of PCR amplification products of genes are listed in Table 2.

**Table 1:** The original number of samples

Number	Sampling number	Origin	Number	Sampling number	Origin	Number	Sampling number	Origin
1	K1	Pig heart	145	K3	Celery	289	K15	Duck
2	K2	Pork	146	K5	Broccoli	290	K16	Duck
3	K4	Pork liver	147	K7	Lettuce	291	K17	Duck leg
4	K6	Pork	148	K11	Tomato	292	K19	Duck
5	K8	Pork	149	K12	Pepper	293	K20	Duck
6	K9	Pork	150	K14	Cabbage	294	K24	Duck
7	K10	Pork stuffing	151	K21	Ginger	295	K25	Duck
8	K13	Porcine blood	152	K22	Celery	296	K27	Duck
9	K18	Pork	153	K23	Pepper	297	K35	Duck
10	K33	Porcine blood	154	K26	Cabbage	298	W7	Duck
11	K34	Pork	155	W1	Broccoli	299	W12	Duck
12	K40	Pork liver	156	W4	Lettuce	300	N4	Fish
13	W2	Pork intestine	157	W5	Pepper	301	N5	Fish
14	W3	Pork liver	158	N1	Ginger	302	N8	Fish
15	W6	Porcine blood	159	N2	Broccoli	303	N14	Fish
16	W8	Pigtail	160	N3	Eggplant	304	N15	Fish
17	W9	Pork	161	S18	Spinach	305	N16	Crustacean
18	W10	Pork fillet	162	S19	Celery	306	N17	Fish
19	W11	Pork liver	163	N6	Shallot	307	W17	Fish
20	W13	Pork	164	N7	Tomato	308	W18	Fish
21	W14	Pork	165	N9	Lettuce	309	W61	Fish
22	W15	Pork	166	W21	Tomato	310	W62	Fish
23	W16	Pork	167	H11	Ginger	311	W63	Fish
24	W19	Pork	168	N52	Cowpea	312	K36	Fish
25	W20	Pork	169	H14	Spinach	313	K37	Fish
26	W25	Porcine blood	170	H15	Broccoli	314	S1	Fish
27	W26	Porcine blood	171	H16	Pepper	315	S2	Fish
28	S5	Pork	172	H17	Shallot	316	S3	Fish
29	S8	Pig heart	173		Tomato	317	S4	Fish
30	S9	Pork stuffing	174	W22	Eggplant	318	W64	Fish
31	S10	Pork fillet	175	W23	Spinach	319	W65	Fish
32	S12	Pork liver	176	W24	Tomato	320	W66	Fish
33	S14	Pig hind leg	177	W67	Celery	321	W69	Fish
34	S15	Pork	178	W68	Ginger	322	W72	Fish
35	S16	Pork liver	179	W70	Shallot	323	W73	Fish
36	S17	Pork	180	W71	Cowpea	324	W75	Fish
37	H2	Pork intestine	181	W74	Tomato	325	W54	Fish
38	H4	Pork	182	W76	Pepper	326	W55	Fish
39	H5	Pork	183	K38	Broccoli	327	W56	Fish
40	H6	Porcine blood	184	K39	Ginger	328	S6	Fish
41	H7	Pig trotters	185	K41	Shallot	329	S7	Fish
42	H8	Porcine blood	186	W77	Lettuce	330	S11	Brine shrimp
43	H9	Pork	187	W78	Cowpea	331	N10	Bean curd skin
44	H12	Porcine blood	188	W79	Spinach	332	N11	Marinated tofu
45	H13	Pork	189	W80	Eggplant	333	N12	Stewed chicken leg
46	H23	Porcine blood	190	S13	Tomato	334	N13	Stewed beef
47	H24	Pork liver	191	H1	Shallot	335	N51	Red oil chicken gizzards
48	H27	Pork	192	H3	Celery	336	K42	Hot and sour gluten
49	H28	Pork	193	H10	Ginger	337	K43	Marinated chicken leg
50	H30	Pork	194	W28	Pepper	338	K45	Cold bamboo shoots
51	H33	Pork	195	W29	Broccoli	339	K74	Soy sauce pickles
52	H34	Pork	196	W34	Tomato	340	K75	Spiced gizzard
53	K28	Celery	197	H66	Lettuce	341	K76	Beef salad
54	K29	Shallot	198	H67	Shallot	342	K77	Beef tendon in cold sauce
55	K30	Spinach	199	H68	Eggplant	343	K78	Cold bamboo shoots
56	N46	Potato	200	H69	Ginger	344	K79	Bean salad
57	N47	Eggplant	201	H70	Spinach	345	S22	Fungus salad
58	N48	Spinach	202	H71	Cowpea	346	S23	Kelp salad
59	N49	Shallot	203	H72	Tomato	347	K80	Bean curd skin in cold sauce
60	W52	Cowpea	204	H73	Coriander	348	K81	Kelp salad
61	W53	Bitter gourd	205	H74	Snow pea	349	W32	Shredded lotus root slice
62	W57	Eggplant	206	H75	Lettuce	350	W33	Spiced gizzard
63	S20	*Flammulina velutipes* mushroom	207	N18	Drumsticks	351	H18	Pea noodles
64	S21	Celery	208	N19	Chicken wings	352	H19	Dried bean curd
65	S24	Zhaer root	209	N20	Drumsticks	353	H20	Bean curd
66	S25	Lettuce	210	N21	Chicken gizzard	354	H26	Red ear silk
67	S26	Chinese cabbage	211	N22	Chicken	355	H29	Chicken salad
68	S27	Bok choy	212	H21	Drumsticks	356	H30	Sweet potato
69	S28	Ginger	213	H22	Chicken wings	357	S95	Chinese wolfberries
70	S47	Tomato	214	K44	Chicken gizzard	358	S96	Cold bean curd
71	S48	Bitter gourd	215	K46	Chicken	359	S97	Bean curd skin
72	S49	Black fungus	216	H23	Chicken wing	360	S98	Gluten
73	S50	Garlic sprouts	217	S53	Drumsticks	361	S99	Cold pig ears
74	S51	Chive	218	N53	Chicken	362	S100	Peanut salad
75	S52	Coriander	219	N54	Chicken wing	363	H76	Cold bamboo shoots
76	N55	Broccoli	220	S64	Drumsticks	364	H77	Marinated tofu
77	N56	Celery	221	S65	Chicken gizzard	365	H78	Spicy dried tofu
78	S61	Pepper	222	S66	Chicken	366	K47	Spicy dried tofu
79	S62	Coriander	223	S67	Drumsticks	367	K64	Red oil ear silk
80	S63	Green Chinese onion	224	S68	Chicken wings	368	K65	Cold bean curd stick
81	H24	Bitter gourd	225	W35	Drumsticks	369	K66	Dried vegetables
82	H25	*Lentinus edodes* mushroom	226	W38	Chicken wings	370	K67	Brine shrimp
83	H27	Pepper	227	S69	Drumsticks	371	K71	Bean curd skin
84	H28	Kelp	228	S70	Chicken gizzard	372	K72	Chicken skewer
85	H31	Pepper	229	S71	Chicken	373	K73	Hot and sour gluten
86	S72	Bean sprouts	230	S29	Chicken wings	374	W36	Marinated tofu
87	S73	*Coprinus comatus* mushroom	231	S30	Chicken	375	W37	Stewed pork liver
88	S74	Romaine lettuce	232	H41	Chicken wings	376	S34	Stewed beef
89	S75	Coriander	233	H42	Drumsticks	377	S35	Stewed chicken leg
90	S76	Tomatoes	234	H43	Drumsticks	378	S36	Marinated tofu
91	S77	Pepper	235	H44	Chicken wings	379	S54	Brine shrimp
92	S78	Celery	236	H60	Chicken gizzard	380	S55	Bean curd skin
93	S79	Lotus root	237	S81	Drumsticks	381	S56	Chicken skewer
94	S80	Cabbage	238	S82	Chicken	382	S57	Marinated chicken leg
95	S89	Cucumber	239	S83	Chicken gizzard	383	N34	Marinated tofu
96	S90	Celery	240	S84	Chicken wings	384	N35	Stewed beef
97	S91	Garlic sprouts	241	S85	Chicken gizzard	385	N36	Stewed beef
98	S92	Spinach	242	S86	Drumsticks	386	N37	Hot and sour gluten
99	S93	Towel gourd	243	S87	Drumsticks	387	N38	Marinated chicken leg
100	S94	Peas	244	S88	Drumsticks	388	N45	Stewed chicken leg
101	K48	Chives	245	K32	Chicken wings	389	N50	Stewed pork liver
102	K49	Garlic sprouts	246	W27	Chicken	390	K61	Marinated tofu
103	K52	Lettuce	247	W30	Drumsticks	391	K62	Stewed pork liver
104	K68	Pepper	248	W31	Chicken wings	392	K63	Lamb tripe
105	K69	Cucumber	249	K53	Chicken	393	K31	Mutton
106	K70	Lettuce	250	K54	Chicken	394	W39	Mutton
107	H40	Cucumber	251	K59	Drumsticks	395	W46	Mutton
108	H45	Pepper	252	K60	Chicken gizzard	396	W51	Sheep heart
109	H48	Peas	253	W47	Chicken gizzard	397	W63	Mutton
110	H50	Cucumber	254	W48	Drumsticks	398	W64	Mutton
111	H56	Lettuce	255	K50	Beef	399	W65	Mutton
112	H57	Towel gourd	256	K51	Beef	400	W66	Mutton
113	H58	Pepper	257	W47	Beef	401	S39	Mutton
114	H59	Peas	258	W48	Beef stuffing	402	S40	Mutton
115	W40	Chives	259	N23	Beef	403	S41	Mutton
116	W43	Spinach	260	N24	Beef	404	S44	Mutton
117	W45	Pepper	261	N25	Beef	405	S58	Mutton
118	W60	Towel gourd	262	N26	Beef	406	S59	Mutton
119	W61	Spinach	263	N27	Beef	407	S60	Mutton
120	W62	Cucumber	264	H32	Beef	408	N31	Mutton
121	S42	Celery	265	H33	Beef	409	N32	Mutton
122	S43	Chives	266	H34	Beef	410	N33	Mutton
123	N28	Peas	267	H61	Beef	411	R1	Retail fresh milk
124	N29	Lettuce	268	H62	Beef	412	R2	Retail fresh milk
125	N30	Pepper	269	H63	Beef	413	R3	Retail fresh milk
126	S31	Towel gourd	270	H64	Beef	414	R4	Retail fresh milk
127	S32	Pepper	271	H65	Beef	415	R5	Retail fresh milk
128	S33	Lettuce	272	W44	Beef stuffing	416	R6	Retail fresh milk
129	W41	Cucumber	273	S37	Beef stuffing	417	R7	Retail fresh milk
130	W42	Peas	274	S38	Beef	418	R8	Retail fresh milk
131	N39	Lettuce	275	S45	Beef	419	R9	Retail fresh milk
132	N40	Lettuce	276	S46	Beef	420	R10	Retail fresh milk
133	K55	Pepper	277	S50	Beef	421	R11	Retail fresh milk
134	K57	Chives	278	S51	Beef	422	R12	Retail fresh milk
135	S47	Towel gourd	279	S53	Beef	423	R13	Retail fresh milk
136	S48	Lettuce	280	K56	Beef	424	R14	Retail fresh milk
137	S52	Cucumber	281	K58	Beef	425	R15	Retail fresh milk
138	N41	Spinach	282	S49	Beef	426	R19	Retail fresh milk
139	N42	Pepper	283	H36	Beef	427	R20	Retail fresh milk
140	N43	Cucumber	284	H37	Beef	428	R21	Retail fresh milk
141	N44	Cucumber	285	W59	Beef	429	R23	Retail fresh milk
142	W49	Chives	286	W60	Beef	430	R26	Retail fresh milk
143	W50	Spinach	287	H38	Beef	431	R31	Retail fresh milk
144	H35	Towel gourd	288	H39	Beef			

Note

H, supermarket sampling in Shihezi; K, samples collected from Kuitun; N, sampling in cooperation with Inspection Institute; R, retail fresh milk collected from Shihezi; S, samples collected from Shihezi; W, samples collected from Urumqi.

**Table 2:** Primers used for detection of genes encoding resistance to different antimicrobials

Gene	Primer	DNA sequence (5′ → 3′)	Size (bp)	Thermocycling conditions	References
*UidA*	*UidA*F	5′‐ATGGAATTTCGCCGATTTTGC‐3′	194	95°C for 5 min, 40 cycles of 95°C for 30 s, 60°C for 1 min, 72°C for 1 min, and final extension at 72°C for 7 min	Heijnen and Medema (2006)
	*UidA*R	5′‐ATTGTTTGCCTCCCTGCTGC‐3′			
*tet*A	*tet*A*‐*F	5′‐GCTACATCCTGCTTGCCTTC‐3′	210	95°C for 5 min, 30 cycles of 94°C for 30 s, 60°C for 1 min, 72°C for 1 min, and final extension at 72°C for 5 min	Ng, Martin, Alfo, and Mulvey (2001)
	*tet*A‐R	5′‐CATAGATCGCCGTGAAGAGG‐3′			Ng et al. (2001)
*tet*B	*tet*B*‐*F	5′‐TTGGTTAGGGGCAAGTTTTG‐3′	659		Ng et al. (2001)
	*tet*B*‐*R	5′‐GTAATGGGCCAATAACACCG‐3′			Ng et al. (2001)
*tet*C	*tet*C*‐*F	5′‐CTTGAGAGCCTTCAACCCAG‐3′	418		Sáenz et al. (2004)
	*tet*C*‐*R	5′‐ATGGTCGTCATCTACCTGCC‐3′			Sáenz et al. (2004)
*bla*_TEM_	*bla*_TEM_‐F	5′‐TTGGGTGCACGACTGGGT‐3′	503	95°C for 5 min, 30 cycles of 94°C for 1 min, 60°C for 1 min, 72°C for 1 min, and final extension at 72°C for 5 min	Knapp, Dolfing, Ehlert, and Graham (2010)
	*bla*_TEM_‐R	5′‐TAATTGTTGCCGGGAAGC‐3′			Knapp et al. (2010)
*bla*_PSE_	*bla*_PSE_ *‐*F	5′‐CGCTTCGGGTTAACAAGTAC‐3′	419		Zhi, Xi, and Shen (2009)
	*bla*_PSE_ *‐*R	5′‐CTGGTTCATTTCAGATAGCG‐3′			Zhi et al. (2009)
*bla*_OXA_	*bla*_OXA_‐F	5′‐AGCAGCGCCAGTGCATCA‐3′	708		Guerra et al. (2003(
	*bla*_OXA_‐R	5′‐ATTCGACCCCAAGTTTCC‐3′			Guerra et al. (2003)
*flo*R	*flo*R‐F	5′‐CACGTTGAGCCTCTATAT‐3′	868	95°C for 5 min, 30 cycles of 94°C for1 min, 52°C for 1 min, 72°C for 1 min, and final extension at 72°C for 10 min	Sáenz et al. (2004)
	*flo*R‐R	5′‐ATGCAGAAGTAGAACGCG‐3′			Sáenz et al. (2004)
*sul*1	*Sul*1‐F	5′‐CGGCGTGGGCTACCTGAACG‐3′	433	94°C for 5 min, 30 cycles of 94°C for 15 s, 69°C for 30 s, 72°C for 1 min, and final extension at 72°C for 7 min	Sáenz et al. (2004)
	*Sul*1‐R	5′‐GCCGATCGCGTGAAGTTCCG‐3′			Sáenz et al. (2004)
*sul*2	*Sul*2‐F	5′‐GCGCTCAAGGCAGATGGCATT‐3′	285		Sáenz et al. (2004)
	*Sul*2‐R	5′‐GCGTTTGATACCGGCACCCGT‐3′			Sáenz et al. (2004)
*aad*_Ala_	*aad*_Ala_ *‐*F	5′‐AACGACCTTTTGGAAACTTCGG−3′	352	94°C for 10 min, 35 cycles of 94°C for 1 min, 60°C for 30 s, 72°C for 1 min, and final extension at 72°C for 10 min	Sáenz et al. (2004)
	*aad*_Ala_ *‐*R	5′‐TTCGCTCATCGCCAGCCCAG‐3′			Sáenz et al. (2004)
*aad*B	*Aad*B*‐*F	5′‐GGGCGCGTCATGGAGGAGTT‐3′	329	94°C for 10 min, 35 cycles of 94°C for 1 min, 65°C for 30 s, 72°C for 1 min, and final extension at 72°C for 10 min	Rosengren, Waldner, and Reid‐Smith (2009)
	*aad*B*‐*R	5′‐TATCGCGACCTGAAAGCGGC‐3′			Rosengren et al. (2009)
*str*A	*Str*A*‐*F	5′‐CCTGGTGATAACGGCAATTC‐3′	546	95°C for 4 min, 35 cycles of 95°C for 1 min, 55°C for 1 min, 72°C for 1 min, and final extension at 72°C for 7 min	Rosengren et al. (2009)
	*Str*A*‐*R	5′‐CCAATCGCAGATAGAAGGC‐3′			Rosengren et al. (2009)
*str*B	*Str*B*‐*F	5′‐ATCGTCAAGGGATTGAAACC‐3′	509		Rosengren et al. (2009)
	*Str*B*‐*R	5′‐GGATCGTAGAACATATTGGC‐3′			Rosengren et al. (2009)

### 2.2 Antimicrobial susceptibility testing

Antimicrobial susceptibility testing was performed utilizing the disk‐diffusion method as recommended by the Clinical and Laboratory Standards Institute (CLSI, 2015). The following antibiotics were used: ampicillin (AMP: 10 μg/p), cefotaxime (CTX: 30 μg/p), ceftazidime (CAZ: 30 μg/p), gentamicin (GEN: 10 μg/p), imipenem (IPM: 10 μg/p), ciprofloxacin (CIP: 5 μg/p), levofloxacin (LEV: 5 μg/p), tetracycline (TET: 30 μg/p), chloramphenicol (CHL: 30 μg/p), amikacin (AMK: 30 μg/p), piperacillin (PIP: 100 μg/p), compound trimethoprim/sulfamethoxazole (T/S: 23.75 μg/1.25 μg/p), erythromycin (ERY: 15 μg/p), amoxicillin (AMX: 10 μg/p), streptomycin (STR: 10 μg/p), nalidixic acid (NAL: 30 μg/p), and polymyxin B (PB: 300 μg/p). Standard strain *E. coli* ATCC 25922 was used as a quality control. Strains were classified as either susceptible, intermediate, or resistant strains (CLSI, 2015).

### 2.3 PCR amplification of antimicrobial resistance and virulence genes

Genomic DNA for PCR was extracted by the boiling method. Tables 2 and 3 list the oligonucleotide sequences of different antimicrobial genes and virulence genes in *E. coli* and the predicted sizes after PCR amplification.

**Table 3:** Primers used for detection of genes encoding resistance to different virulence

Gene	Primer	DNA sequence (5′ → 3′)	Size (bp)	Thermocycling conditions	References
*stx*1	*stx*1*‐*F	5′‐ACACTGGATGATCTCAGTGG‐3′	244	95°C for 5 min, 35 cycles of 94°C for 1 min, 60°C for 1 min, 72°C for 1 min, final extension at 72°C for 10 min	Moses, Garbati, and Egwu (2006)
	*stx*1*‐*R	5′‐CTGAATCCCCCTCCATTATG‐3′			Moses et al. (2006)
*stx*2	*stx*2‐F	5′‐CCATGACAACGGACAGCAGTT‐3′	255		Moses et al. (2006)
	*stx*2‐R	5′‐CCTGTCAACTGAGCACTTTG‐3′			Moses et al. (2006)
*agg*	*agg*‐F	5′‐AAGAAAAAGAAGTAGACCAAC‐3′	400		Pass, Odedra, and Batt (2000)
	*agg*‐R	5′‐AAACGGCAAGACAAGTAAATA‐3′			Pass et al. (2000)
*eae*A	*eae*‐F	5′‐AAGCGACTGAGGTCACT‐3′	384		Lopez et al. (2003)
	*eae*‐R	5′‐ACGCTGCTCACTAGATGT‐3′			Lopez et al. (2003)
*fyu*A	*fyu‐*F	5′‐ACACGGCTTTATCCTCTGGC‐3′	235	95°C for 5 min, 30 cycles of 94°C for 30 s, 52°C for 30 s, 72°C for 45 s, and final extension at 72°C for 10 min	Viktoria, Lionel, and Per (2008)
	*fyu‐*R	5′‐GGCATATTGACGATTAACGA‐3′			Viktoria et al. (2008)
*fim*A	*fim*A*‐*F	5′‐CTGTGAGTGGTCAGGCAAGCG‐3′	352		Rawool et al. (2015)
	*fim*A*‐*R	5′‐TAACCGTGTTGGCGTAAGAGC‐3′			Rawool et al. (2015)
*pap*C	*pap*C‐F	5′‐GACGGCTGTACTGCAGGGTCGGGCG‐3′	234	95°C for 5 min, 30 cycles of 94°C for 30 s, 47°C for 30 s, 72°C for 45 s, and final extension at 72°C for 10 min	Xia et al. (2011)
	*pap*C‐R	5′‐ATATCCTTTCTGCAGGGATGCAATA‐3′			Xia et al. (2011)
*pap*A	*pap*A‐F	5′‐GGAACGAACGCAGAAACG‐3′	374	95°C for 5 min, 30 cycles of 94°C for 30 s, 52°C for 30 s, 72°C for 45 s, and final extension at 72°C for 10 min	Xia et al. (2011)
	*pap*A‐R	5′‐CGCAATGGGCGAATACTT‐3′			Xia et al. (2011)
*fim*C	*fim*C‐F	5′TAAGGAAATCGCAGGAA‐3′	337	95°C for 5 min, 30 cycles of 94°C for 30 s, 50°C for 30 s, 72°C for 45 s, and final extension at 72°C for 10 min	Antonio et al. (2007)
	*fim*C‐R	5′‐GCTGTGGGATAATGGACT‐3′			Antonio et al. (2007)

The presence of genes associated with resistance to tetracycline (*tet*A, *tet*B, and *tet*C), β‐lactams (*bla*_TEM_, *bla*_PSE_, and *bla*_OXA_), aminoglycosides (*aad*_A1a_, *aad*B, *str*A, and *str*B), chloramphenicol (*flo*R), and sulfonamide (*Sul*1 and *Sul*2), and virulence‐encoding genes were detected by PCR. The PCR products were electrophoresed for 40 min at 90 V in 1% agarose gel containing 0.5 µg/ml of ethidium bromide, and then, the gels were visualized on a Gel Doc 2000 transmittance apparatus (Kerrn, Klemmensen, Frimodt‐MØller, & Espersen, 2002). Target fluorescentbands were removed from the gel with a razor blade. The DNA fragments were purified with a MIDI gel purification kit and then sequenced. The DNA sequence data were compared with the data in the GenBank database.

### 2.4 Statistical analysis

SPSS v.17.0 software was used to analyze the data. Logistical regression analysis was used to analyze the correlation between variables. *p* < .05 was considered statistically significant.

## 3 RESULTS AND CONCLUSIONS

### 3.1 *E. coli *isolated from retail foods

A total of 112 strains of *E. coli* were isolated from 431 random samples, with 26% of the samples testing positive for contamination. The overall incidence was higher than 14.7% reported elsewhere (Rasheed et al., 2014). As shown in Table 4, pork was most frequently contaminated with *E. coli* (59.6%). The detection rates of *E. coli* were 52.6%, 52.4%, 36.4%, 35.3%, and 33.3% in mutton, retail fresh milk, duck, beef, and chicken, respectively, followed by ready‐to‐eat food (12.9%), vegetables (11%), and fish (10%).

**Table 4:** Samples and isolates from different food origins

Products	No. of samples	No. of samples positive for *E. coli*	Positive rate (%)
Pork	52	31	59.6
Chicken	48	16	33.3
Duck	11	4	36.4
Fish	30	3	10.0
Retail fresh milk	21	11	52.4
Beef	34	12	35.3
Mutton	19	10	52.6
Vegetables	154	17	11.0
Ready‐to‐eat food	62	8	12.9
Total	431	112	26.0

Several studies have documented antibiotic‐resistant *E. coli *and other coliforms in raw meat (Srinivasa, Gill, Ravi, & Sandeep, 2011), poultry (Nuno et al., 2016), eggs (Arathy, Vanpee, Belot, DeAllie, & Sharma, 2011), milk (Alharbi & Khaled, 2018), and vegetables (Rasheed et al., 2014). Whether there is a link between high contamination rates and high antibiotic resistance rates for *E. coli* in food remains to be determined.

In both developed and developing countries, antibiotic resistance has been recognized as a problem in the field of human and veterinary medicine (Bottacini et al., 2018; Zhang et al., 2017). There is ample evidence that the widespread use of antibiotics in agriculture and medicine is the main reason for the high resistance rate of Gram‐negative bacteria (Bothyna & Randa, 2018). Various food and environmental sources contain bacteria resistant to one or more antimicrobial agents used in human or veterinary medicine and animal food production (Hinthong, Pumipuntu, & Santajit, 2017).

### 3.2 Antimicrobial resistance profiles of *E. coli *isolates

Antibiotic resistance in *E. coli* is of particular concern because it is the most common Gram‐negative pathogen in humans, the most common cause of urinary tract infections, and a frequent cause of community and hospital‐acquired bacteremia (Bothyna & Randa, 2018) and diarrhea (Jessica, Lashaunda, & Levens, 2016).

Worldwide data have shown that resistance to traditional drugs is increasing, and resistance is also being encountered against newer and more effective antibiotics (Sara, Mohammad, & Sadegh, 2014). As in this study, the most frequent resistance was seen for third‐generation cephalosporin–ceftazidime (22%) and tetracyclines (52%; Table 5). A comparative study by Dominguez et al. (2018) showed that high resistance rates (76.5%–79.4%) were observed in oxyimino‐cephalosporins (cefotaxime, ceftriaxone, and ceftiofur) and cefepime (70.6%). This phenomenon requires additional study and sustained data support.

**Table 5:** The reactions of *E. coli* to 17 antibacterial agents

Antimicrobials	Resistant (*n* = 112）	Susceptible (*n* = 112, %)
AMP	47 (42%)	23 (20)
CTX	12 (11%)	34 (30)
CAZ	25 (22%)	38 (34)
IPM	0	112 (100)
PIP	31 (28%)	40 (36)
AMX	37 (33%)	35 (31)
PB	2 (2%)	72 (64)
CIP	18 (16%)	48 (43)
LEV	12 (11%)	50 (45)
NAL	36 (32%)	34 (30)
GEN	12 (11%)	50 (45)
AMK	10 (9%)	55 (49)
STR	24 (21%)	44 (39)
TET	58 (52%)	22 (20)
CHL	30 (27%)	38 (34)
T/S	41 (37%)	32 (29)
ERY	12 (11%)	38 (34)

Note

*n* = 112: No. of samples positive for *E. coli*.

As shown in Table 5, our study revealed that 87 (77.7%) isolates (*n* = 112) were resistant to one or more antimicrobials, including tetracycline (52%), ampicillin (42%), compound trimethoprim/sulfamethoxazole (37%), amoxicillin (33%), and nalidixic acid (32%). No resistance to imipenem was observed. Among those isolates, two strains (E36, E37) isolated from chicken and one strain (E38) isolated from mutton were resistant to 13 antimicrobial agents. There were two strains (E24 and E53) isolated from chicken and one strain (E56) isolated from fish resistant to 11 antimicrobial agents. The specific multiple drug resistance rate is shown in Table 6, and the pattern of antibiotic resistance in those isolates is shown in Table 7.

**Table 6:** Profile of multiple antibiotic‐resistant *Escherichia coli* isolates

Resistance type													The number of multi‐drug‐resistant strain	The rate of multi‐drug‐resistant strains (%; *n* = 112)
AMP	CTX	GEN	CIP	LEV	TET	CHL	AMK	PIP	T/S	AMX	STR	NAL	E36	3 (2.7)
AMP	CTX	CAZ	GEN	CIP	LEV	TET	CHL	AMK	PIP	T/S	AMX	NAL	E37	
AMP	CTX	CAZ	GEN	CIP	LEV	TET	CHL	AMK	PIP	T/S	AMX	NAL	E38	
CAZ	CIP	LEV	TET	CHL	PIP	T/S	ERY	AMX	STR	NAL			E24	3 (2.7)
CTX	GEN	TET	CHL	AMK	PIP	T/S	ERY	AMX	STR	NAL			E53	
AMP	CTX	CAZ	CIP	LEV	TET	T/S	ERY	AMX	STR	NAL			E56	
AMP	CTX	GEN	CIP	TET	STR	AMK	PIP	AMX	T/S				F41	1 (0.9)
AMP	CTX	CAZ	CIP	TET	CHL	T/S	ERY	NAL					E48	1 (0.9)
AMP	CAZ	TET	CHL	PIP	T/S	AMX	CIP						E28	5 (4.5)
AMP	CAZ	TET	CHL	AMK	T/S	ERY	AMX						E31	
AMP	CAZ	TET	CHL	PIP	T/S	ERY	LEV						E42	
AMP	TET	T/S	CAZ	CHL	AMX	STR	NAL						E47	
AMP	CIP	LEV	TET	T/S	AMX	STR	NAL						F38	
AMP	TET	PIP	T/S	ERY	AMX	NAL							E9	6 (5.4)
AMP	CAZ	GEN	PIP	T/S	AMX	AMK							E23	
AMP	CAZ	TET	PIP	AMX	CIP	LEV							E41	
CAZ	TET	CHL	T/S	AMX	STR	NAL							E46	
CAZ	TET	PIP	T/S	AMX	STR	NAL							E49	
TET	NAL	T/S	AMP	PIP	AMX	CHL							F21	
AMP	CIP	TET	CHL	PIP	T/S								E2	12 (11)
AMP	TET	CHL	PIP	T/S	AMX								E6	
AMP	CTX	CAZ	PIP	NAL	PB								E22	
AMP	CTX	CAZ	TET	PIP	T/S								E32	
AMP	CAZ	TET	PIP	NAL	CHL								E34	
AMP	CAZ	TET	CHL	T/S	AMX								E44	
AMP	TET	CHL	PIP	T/S	AMX								E52	
AMP	CTX	CAZ	TET	T/S	NAL								E54	
AMP	TET	CHL	AMK	T/S	NAL								E55	
TET	NAL	T/S	AMP	PIP	AMX								F1	
TET	NAL	T/S	AMP	PIP	AMX								F3	
TET	NAL	T/S	AMP	PIP	AMX								F11	
TET	CHL	T/S	NAL	CIP									E5	11 (10)
AMP	TET	CHL	T/S	STR									E8	
AMP	TET	PIP	AMX	NAL									E43	
GEN	TET	CHL	T/S	AMX									E51	
NAL	T/S	AMP	LEV	CHL									F10	
TET	NAL	AMP	PIP	LEV									F18	
TET	AMP	PIP	AMX	CHL									F19	
TET	NAL	T/S	AMP	LEV									F24	
AMP	PIP	AMX	CHL	STR									F30	
TET	NAL	T/S	GEN	STR									F32	
NAL	PIP	AMX	STR	ERY									F56	
GEN	CIP	TET	AMX										E3	9 (8)
AMP	TET	CHL	T/S										E12	
CAZ	TET	AMX	STR										E19	
CIP	ERY	AMX	NAL										E20	
TET	NAL	PIP	AMK										E26	
CAZ	TET	AMX	NAL										E27	
TET	T/S	CIP	AMK										E33	
TET	AMP	PIP	STR										F45	
TET	NAL	AMP	STR										F47	
CAZ	TET	CIP											E18	10 (9)
CTX	CAZ	CHL											E39	
TET	AMX	CHL											E40	
AMP	CTX	CAZ											E45	
TET	T/S	AMP											F9	
CHL	STR	ERY											F23	
TET	NAL	AMP											F35	
T/S	AMX	STR											F49	
CHL	ERY	STR											F53	
CHL	GEN	STR											F55	
TET	T/S												E1	16 (14)
AMP	CAZ												E15	
AMP	CIP												E16	
CAZ	NAL												E17	
AMP	TET												E21	
PB	CIP												E25	
AMP	AMX												F4	
AMP	PIP												F6	
AMP	PIP												F15	
AMP	STR												F17	
TET	STR												F28	
TET	NAL												F29	
NAL	T/S												F31	
AMP	GEN												F39	
GEN	STR												F42	
TET	STR												F44	

**Table 7:** Phenotypic and genotypic resistance patterns of *E. coli* isolates

Sampling number	Origin	Strain number	Resistance to antimicrobial agent	Resistance gene(s)
K2	Pork	E1	TET‐T/S	*tet*A, *bla*_OXA_, *bla*_TEM_
K13	Pork tenderloin	E2	AMP‐CIP‐TET‐CHL‐PIP‐T/S	*tet*A, *flo*R
N19	Chicken wings	E3	GEN‐CIP‐TET‐AMX	*tet*A
K50	Beef	E4	—	*bla*_OXA_, *flo*R
K34	Pork	E5	TET‐CHL‐T/S‐NAL‐CIP	*tet*A, *bla*_OXA_, *flo*R, *aad*_Ala_, *Sul*1
K46	Chicken	E6	AMP‐TET‐CHL‐PIP‐T/S‐AMX	*bla*_OXA_ *,bla*_TEM_ *,, Sul*1, s*ul*2, *str*B
K51	Beef	E7	—	*aad*B
K17	Duck leg	E8	AMP‐TET‐CHL‐T/S‐STR	*flo*R, *Sul*1, s*ul*2, *str*A, *str*B
S24	Zhaer root leaf vegetable	E9	AMP‐TET‐PIP‐T/S‐ERY‐AMX‐NAL	*tet*A, *flo*R, *Sul*1, *str*A
S99	Cold pig ears	E10	—	—
S100	Peanut salad	E11	—	—
H8	Porcine blood	E12	AMP‐TET‐CHL‐T/S	*aad*B, *str*A
H22	Chicken wings	E13	—	—
W41	Mutton	E14	—	*str*A
N23	Beef	E15	AMP‐CAZ	—
S25	Lettuce	E16	AMX‐CIP	*str*A
K14	Chinese cabbage	E17	CAZ‐NAL	*tet*A
H23	Chicken wings	E18	CAZ‐TET‐CIP	*tet*A
H76	Cold bamboo shoots	E19	CAZ‐TET‐AMX‐STR	*tet*B, *Sul*1, s*ul*2, *str*A, *str*B
S65	Chicken breast	E20	CIP‐ERY‐AMX‐NAL	*str*A
S49	Black fungus	E21	AMP‐TET	*tet*A
H32	Beef	E22	AMP‐CTX‐CAZ‐PIP‐NAL‐PB	*tet*A, *bla*_OXA_, *bla*_TEM_,
W9	Pork	E23	AMP‐CAZ‐GEN‐PIP‐T/S‐AMX‐AMK	*tet*B, *bla*_OXA_, *aad*_Ala_
S55	Chicken wings	E24	CAZ‐CIP‐LEV‐TET‐CHL‐PIP‐T/S‐ERY‐AMX‐STR‐NAL	*flo*R, *Sul*1, s*ul*2, *aad*_Ala_, *str*A, *str*B
H33	Beef	E25	PB‐CIP	*tet*A, *bla*_OXA_, *str*A
W39	Mutton	E26	TET‐NAL‐PIP‐AMK	*tet*A, *tet*B, *aad*B
W46	Mutton	E27	CAZ‐TET‐AMX‐NAL	*bla*_TEM_, *str*A
K4	Pork liver	E28	AMP‐CAZ‐TET‐CHL‐PIP‐T/S‐AMX‐CIP	*tet*A, *bla*_OXA_, *flo*R, *sul*2, *aad*_Ala_, *str*A,*str*B
H65	Beef hind legs	E29	AMX	—
H61	Dried beef	E30	—	*bla*_TEM_
H13	Pork	E31	AMP‐CAZ‐TET‐CHL‐AMK‐T/S‐ERY‐AMX	*bla*_OXA_, *flo*R, *aad*_Ala_
N11	Marinated tofu	E32	AMP‐CTX‐CAZ‐TET‐PIP‐T/S	*bla*_TEM_
S66	Chicken	E33	TET‐T/S‐CIP‐AMK	*tet*A, *aad*_Ala_
H27	Pork	E34	AMP‐CAZ‐TET‐PIP‐NAL‐CHL	*flo*R*, bla*_OXA_
K47	Spicy dried tofu	E35	TET	*tet*A, *tet*B
W38	Chicken wings	E36	AMP‐CTX‐GEN‐CIP‐LEV‐TET‐CHL‐AMK‐PIP‐T/S‐AMX‐STR‐NAL	*bla*_TEM_, *bla*_OXA_, *flo*R, *sul*2, *str*A, *str*B, *tet*A
S70	Chicken gizzard	E37	AMP‐CTX‐CAZ‐GEN‐CIP‐LEV‐TET‐CHL‐AMK‐PIP‐T/S‐AMX‐NAL	*tet*A, *tet*B, *flo*R, *sul*2, *str*A, *str*B
S39	Mutton	E38	AMP‐CTX‐CAZ‐GEN‐CIP‐LEV‐TET‐CHL‐AMK‐PIP‐T/S‐AMX‐NAL	*aad*B, *tet*A, *tet*B
K40	Pork liver	E39	CTX‐CAZ‐CHL	*bla*_OXA_
W2	Pork	E40	TET‐AMX‐CHL	*tet*A, *bla*_TEM_
S71	Chicken	E41	AMP‐CAZ‐TET‐PIP‐AMX‐CIP‐LEV	*tet*B, *bla*_OXA_, s*ul*2, *aad*B, *str*A, *str*B
H24	Pork liver	E42	AMP‐CAZ‐TET‐CHL‐PIP‐T/S‐ERY‐LEV	*tet*A, *tet*B, *bla*_OXA_
H60	Chicken gizzard	E43	AMP‐TET‐PIP‐AMX‐NAL	*tet*A, *tet*B, *bla*_TEM_
K33	Porcine blood	E44	AMP‐CAZ‐TET‐CHL‐T/S‐AMX	*tet*A, *bla*_TEM_,*flo*R
H78	Spicy dried tofu	E45	AMP‐CTX‐CAZ	*tet*A
H28	Pork liver	E46	CAZ‐TET‐CHL‐T/S‐AMX‐STR‐NAL	*tet*A, *bla*_TEM_, *Sul*1, s*ul*2, *aad*B, *str*A, *str*B
H30	Pork	E47	AMP‐TET‐T/S‐CAZ‐CHL‐AMX‐STR‐NAL	*tet*A, *tet*B, *Sul*1, s*ul*2, *str*B
H34	Pork liver	E48	AMP‐CTX‐CAZ‐CIP‐TET‐CHL‐T/S‐ERY‐NAL	*tet*A, *tet*B, *Sul*1, s*ul*2, *str*A, *str*B
S10	Pork fillet	E49	CAZ‐TET‐PIP‐T/S‐AMX‐STR‐NAL	*tet*A, *Sul*1, s*ul*2, *str*A, *str*B
N31	Mutton	E50	—	*bla*_TEM_
K10	Pork stuffing	E51	GEN‐TET‐CHL‐T/S‐AMX	*tet*A, *bla*_TEM_
W3	Pork liver	E52	AMP‐TET‐CHL‐PIP‐T/S‐AMX	*tet*A, *tet*B, *bla*_TEM_, *aad_Ala_*
S30	Chicken	E53	CTX‐GEN‐TET‐CHL‐AMK‐PIP‐T/S‐ERY‐AMX‐STR‐NAL	*tet*A, *tet*B, *bla*_TEM_, *Sul*1, s*ul*2, *str*A, *str*B
H64	Beef hind legs	E54	AMP‐CTX‐CAZ‐TET‐T/S‐NAL	*tet*A, *tet*B, *str*A, *str*B
K64	Red oil ear silk	E55	AMP‐TET‐CHL‐AMK‐T/S‐NAL	s*ul*2
N5	Fish	E56	AMP‐CTX‐CAZ‐CIP‐LEV‐TET‐T/S‐ERY‐AMX‐STR‐NAL	*bla*_TEM_, *str*A, *str*B, s*ul*1, s*ul*2, *str*B
N16	Crustacean	F1	TET‐NAL‐T/S‐AMP‐PIP‐AMX	*str*A, *str*B, *bla*_OXA_, *tet*A, *flo*R, *Sul*1, s*ul*2
R1	Retail fresh milk	F2	—	*tet*B
S27	Bok choy	F3	TET‐NAL‐T/S‐AMP‐PIP‐AMX	*str*A, *str*B, s*ul*2, *bla*_OXA_, *tet*A, *bla*_TEM_, *aad*_Ala_, *flo*R
S56	Broccoli	F4	AMP‐AMX	*tet*B
S96	Cold bean curd stick	F5	—	_—_
W51	Sheep heart	F6	AMP‐PIP	*str*A, *str*B, *bla*_TEM_, *aad*_Ala_, *flo*R, *Sul*1, s*ul*2
S72	Bean sprouts	F7	TET	*bla*_OXA_
H4	Pork	F8	TET	*str*A, *str*B, s*ul*2, *bla*_OXA,_, *tet*A, *bla*_TEM_
H9	Pork	F9	TET‐T/S‐AMP	*tet*A
N22	Chicken	F10	NAL‐T/S‐AMP‐LEV‐CHL	*str*B, *aadA1a*, *flo*R, *Sul*1, s*ul*2
R2	Retail fresh milk	F11	TET‐NAL‐T/S‐AMP‐PIP‐AMX	*bla*_OXA_
N30	Pepper	F12	—	*—*
W8	Pig tail	F13	T/S	*bla*_OXA_, *tet*B, *aad*_Ala_
R5	Retail fresh milk	F14	T/S	*tet*B
R7	Retail fresh milk	F15	AMP‐PIP	*flo*R
R8	Retail fresh milk	F16	—	*bla*_OXA_, *aad*B
S38	Beef	F17	AMP‐STR	*str*B, s*ul*2, *bla*_OXA_
K44	Chicken gizzard	F18	TET‐NAL‐AMP‐PIP‐LEV	*bla*_OXA_
W47	Beef	F19	TET‐AMP‐PIP‐AMX‐CHL	*str*A, *str*B, s*ul*2, *bla*_OXA_, *aad*_Ala_
R8	Retail fresh milk	F20	—	*bla*_OXA_
H9	Pork	F21	TET‐NAL‐T/S‐AMP‐PIP‐AMX‐CHL	*str*A, *str*B, s*ul*2, *bla*_OXA_, *tet*A, *tet*B, *bla*_TEM_, *flo*R, *aad*B
K28	Celery	F22	—	*bla*_OXA,_
H33	Pork	F23	CHL‐STR‐ERY	*str*A, *str*B, *bla*_OXA_, *aad*_Ala_, *Sul*1, s*ul*2, *aad*B
S68	Chicken wings	F24	TET‐NAL‐T/S‐AMP‐LEV	*str*A, *str*B, *Sul*1, s*ul*2_,_, *tet*A, *bla*_TEM_, *aad*_Ala_
S79	Lotus root	F25	ERY	*tet*B
S80	Cabbage	F26	—	*bla*_OXA_
S89	Cucumber	F27	TET	*bla*_OXA_, *tet*A, *tet*B
S58	Sheep fat	F28	TET‐STR	*bla*_OXA_, *tet*B, *aad*_Ala_
K60	Chicken gizzard	F29	TET‐NAL	*tet*B
S8	Pig heart	F30	AMP‐PIP‐AMX‐CHL‐STR	*str*A, *str*B, *bla*_OXA_, *tet*A, *bla*_TEM_, *aad*_Ala_, *Sul*1
W13	Pork	F31	NAL‐T/S	*bla*_OXA_
W14	Pork	F32	TET‐NAL‐T/S‐GEN‐STR	*bla*_TEM_, *aad*_Ala_, *aad*B
K26	Carrot	F33	—	—
R9	Retail fresh milk	F34	—	s*ul*2, *bla*_OXA_
S60	Mutton	F35	TET‐NAL‐AMP	*tet*A, *tet*B, *bla*_OXA_
H34	Beef	F36	—	—
R3	Retail fresh milk	F37	—	*bla*_OXA_
S59	Lamb tripe	F38	AMP‐CIP‐LEV‐TET‐T/S‐AMX‐STR‐NAL	*bla*_OXA_, *tet*B, *flo*R
R6	Retail fresh milk	F39	AMP‐GEN	*bla*_OXA_, *tet*A, *aad*_Ala_, *flo*R
R7	Retail fresh milk	F40	—	—
S90	Celery	F41	AMP‐CTX‐GEN‐CIP‐TET‐STR‐AMK‐PIP‐T/S‐AMX	*str*A, *str*B, s*ul*2, *tet*A, *tet*B, *aad*_Ala_, *flo*R
R10	Retail fresh milk	F42	GEN‐STR	*bla*_OXA_, *aad*B
S45	Beef	F43	NAL	*bla*_OXA_
S12	Pork liver	F44	TET‐STR	*bla*_OXA_, *tet*A, *tet*B, *aad*_Ala_
S41	Lamb tripe	F45	TET‐AMP‐PIP‐STR	*bla*_OXA_, *tet*B
K66	Dried vegetables	F46	—	*tet*B
S91	Garlic sprouts	F47	TET‐NAL‐AMP‐STR	*tet*A, *tet*B, *bla*_OXA_
K32	Chicken wings	F48	—	*bla*_OXA_
W43	Spinach	F49	T/S‐AMX‐STR	s*ul*2
H12	Porcine blood	F50	—	—
N10	Bean curd skin	F51	—	*bla*_OXA_
S93	Towel gourd	F52	—	—
K19	Duck	F53	CHL‐ERY‐STR	*flo*R,*aad*B
K25	Duck	F54	LEV	s*ul*2
W12	Duck	F55	CHL‐GEN‐‐STR	s*ul*2, *aad*_Ala_
N4	Fish	F56	NAL‐PIP‐AMX‐STR‐ERY	*str*A

Note

—, not detected.

The incidence of multidrug resistance is a compelling issue, as there is a repository of antimicrobial resistance genes in the community, and drug resistance genes and plasmids can easily be transferred to other strains. The high resistance to tetracycline and ampicillin may be due to the easy availability and low cost of those medications. Although these antibiotics have been banned, the bans have not been effectively implemented by the relevant regulatory bodies. Another explanation for a strain's high resistance rate is its contact with environmental microorganisms that produce natural antibiotics, or with soil contaminated by wildlife feces carrying antibiotic‐resistant microorganisms.

### 3.3 Antimicrobial resistance genotypes of *E. coli* isolates

We detected 11 of the 13 resistance genes (*tet*A, *tet*B, *bla*_tem_, *bla*_oxa_, *flo*R, *aad*_Ala_, *aad*B, *sul*1, *sul*2, *str*A, and *str*B), and one hundred isolates carried one or more antimicrobial genes. Resistance genes were not detected in twelve strains of *E. coli*. The resistance genotypes of *E. coli* isolates are shown in Table 7.

Among 58 tetracycline‐resistant *E. coli* isolates, *tet*A was found in 43 isolates and *tet*B in 30 isolates, although *tet*C was not detected in any. One of the beta‐lactam resistance genes, *bla*_TEM_, was detected in 23 *E. coli* isolates, *bla*_OXA_ was detected in 45, and *bla*_PSE_ was not detected. Other resistance genes such as *flo*R, s*ul*1, s*ul*2, *aad*_Ala_, *aad*B, s*tr*A, and *str*B were detected in 22, 18, 30, 21, 12, 31, and 27 isolates, respectively. The detection rate of resistance genes of our study was as follows: *tet*A (38%, 43/112), *tet*B (27%, 30/112), *bla*_OXA_ (40%, 45/112), *bla*_TEM _(20%, 23/112), *flo*R (20%, 22/112), *sul*1 (16%, 18/112), *sul*2 (27%, 30/112), *aad*_Ala_ (19%, 21/112), *aad*B (11%, 12/112), *str*A (28%, 31/112), and *str*B (24%, 27/112). These data suggest that retail foods may be a reservoir of multi‐drug‐resistant bacteria and contribute to the spread of drug‐resistant genes.

We found that the detection rate of pork was more than that of chicken, duck, and beef, but there are fewer resistance genes in pork as compared to chicken. Ayoyi, Bii, and Okemo (2008) showed that multidrug resistance is closely related to different farm management treatments, and statistical significance (*p* ≤ .001) was found between them.

Chickens are more likely to get sick than pigs, and in large‐scale chicken breeding operations, farmers will use a large number of antibiotic and antiviral drugs for the prevention and treatment of chicken diseases. The antibiotics used include enrofloxacin, amikacin, colistin, ciprofloxacin, azithromycin, doxycycline hydrochloride, levofloxacin, lincomycin, doxycycline, gentamicin, gentamicin, levofloxacin, neomycin sulfate, ceftriaxone sodium, cefotaxime sodium, penicillin, sulfachloropyridine, and sulfaquinoxaline sodium.

### 3.4 Virulence genes of *E. coli* isolates

Table 8 shows that among the nine tested virulence genes, *fim*C, *agg*, *stx*2, *fim*A, *fyu*A, *pap*A, *stx*1, and *eae*A were found in 52, 34, 21, 19, 6, 3, 2, and 2 isolates, respectively, *pap*C was not detected. Two strains (F6, F52) carried five virulence genes, and six strains (F5, F11, F12, F14, F50, and F51) also carried four virulence genes. Detailed results are shown in Table 9.

**Table 8:** The detection rate of strains and virulence genes

Virulence genes	No. of positive strains	Number of positive strains	Positive rate (%; *n* = 112)
*stx*1	F1, F11	2	1.8
*stx*2	F3, F4, F5, F6, F7, F11, F12, F14, F17, F18, F20, F29, F36, F39, F45, F47, F48, F49, F50, F51, F52	21	18.8
*eae*A	F6, F18	2	1.8
*agg*	E2, E7, E13, E14, E24, E39, F1, F5, F6, F8, F10,F11,F12, F16, F17, F18, F19, F21, F22, F24, F27, F28, F29, F32, F33, F34, F37, F38, F43, F44, F49, F50, F51, F52	34	30.4
*fyu*A	E6, E13, E53, F13, F14, F50	6	5.4
*pap*A	E24, F14, F52	3	2.7
*pap*C	—	0	0
*fim*A	E5、E23、E26、E29、E33、E50, F2, F3, F5, F6, F10, F11, F12, F24, F25, F28, F50, F51, F52	19	17.0
*fim*C	E4, E5, E6, E7, E8, E12, E22, E24, E26, E28, E29, E30, E35, E38, E43, E45, E49, E52, E54, E56, F1, F2, F3, F4, F5, F6, F8, F12, F13, F14, F17, F19, F22, F23, F24, F25, F27, F28, F30, F31, F33, F34, F35, F36, F37, F38, F43, F45, F47, F49, F51F52	52	46.4

**Table 9:** Profile of *Escherichia coli* isolates with multiple virulence genes

Virulence genes					No. of strains with multiple virulence genes	The rate of strains with multiple virulence genes (%; *N* = 112)
*Stx*2	*agg*	*pap*A	*fim*A	*fim*C	F52	2 (1.8)
*Stx*2	*agg*	*eae*A	*fim*A	*fim*C	F6	
*Stx*1	*Stx*2	*agg*	*fim*A		F11	6 (5.4)
*Stx*2	*fyu*A	*pap*A	*fim*C		F14	
*Stx*2	*agg*	*fim*A	*fim*C		F51	
*Stx*2	*agg*	*fim*A	*fim*C		F5	
*Stx*2	*agg*	*fim*A	*fim*C		F12	
*Stx*2	*agg*	*fim*A	*fyu*A		F50	
*Stx*1	*agg*	*fim*C			F1	7 (6.3)
*Stx*2	*fim*A	*fim*C			F5	
*Stx*2	*agg*	*fim*C			F12	
*agg*	*fim*A	*fim*C			F24	
*agg*	*fim*A	*fim*C			F28	
*Stx*2	*agg*	*fim*C			F49	
*Stx*2	*eae*A	*agg*			F18	
*Stx*2	*fim*C				F4	23 (20.5)
*Stx*2	*agg*				F18	
*Stx*2	*fim*C				F36	
*Stx*2	*fim*C				F45	
*Stx*2	*fim*C				F47	
*agg*	*fim*C				E7	
*agg*	*fim*C				E24	
*agg*	*fim*C				F8	
*agg*	*fim*C				F19	
*agg*	*fim*C				F22	
*agg*	*fim*C				F27	
*agg*	*fim*C				F33	
*agg*	*fim*C				F34	
*agg*	*fim*C				F37	
*agg*	*fim*C				F38	
*agg*	*fim*C				F43	
*agg*	*fim*A				E7	
*fyu*A	*fim*C				E6	
*fyu*A	*fim*C				F13	
*fim*A	*fim*C				E5	
*fim*A	*fim*C				E26	
*fim*A	*fim*C				E29	
*fim*A	*fim*C				F2	

The emergence of virulence is mainly due to the presence of multiple virulence genes in *E. coli* pathogenicity islands. *fyu*A is highly pathogenic and is often used as an indication of the presence or absence of high pathogenicity islands (HPI; Paniagua et al., 2017). We detected *fyu*A virulence genes in six isolates (5.4%), compared to 83.3% found by Laupland, Gregson, Church, Ross, and Pitout (2008).

Bacterial pili and fimbriae are important structures for bacterial pathogenicity, and it has been suggested that type I fimbriae function primarily in the initial pathogenic phase of avian pathogenic *E. coli* (APEC) infection. P‐type fimbriae are also thought to contribute to bacterial pathogenicity (Paniagua et al., 2017). The *fim*C virulence gene encodes a protein necessary for the biosynthesis of type I fimbriae. The *pap*A virulence gene encodes the main protein component of P‐type fimbriae, and P‐type fimbriae are encoded by the nine‐gene *pap* operon, which includes *pap*A, *pap*B, *pap*C, *pap*D, *pap*E, *pap*F, *pap*G, *pap*H, and *pap*I. Sequence analysis showed that there is sufficienthomology between P fimbriae in humans and chickens to indicate that they share some common antigen (Laupland, Kibsey, & Gregson, 2013). We detected the *fim*C gene in 46.4% of isolates, and the *pap*A gene was detected in 2.7%; *pap*C was not detected. This suggests that APEC in the Xinjiang region is mainly caused by a type I fimbriae.

### 3.5 The relationship between virulence genes and antibiotic resistance

Arisoy et al. (2008) showed that there was a correlation between antibiotic sensitivity and virulence factors (VFs) of *E. coli* isolates causing pyelonephritis. They reported an increased presence of virulence genes *pap*, *sfa*, *afai*, *hly*, and *aer *in sensitive strains. Horcajada et al. (2005) showed that a significant correlation was found between nalidixic acid resistance and the decreased prevalence of three VFs: *sfa*, *hly*, and *cnf‐1*.

In the current study, strong associations were found between the presence of *fim*C and resistance to ciprofloxacin, gentamicin, amikacin, levofloxacin, and streptomycin; between the presence of *fim*A and resistance to tetracycline, ampicillin, compound trimethoprim/sulfamethoxazole, and amoxicillin; between the presence of *agg *and resistance to gentamicin, tetracycline, ciprofloxacin, and levofloxacin; and between the presence of *stx*2 and resistance to ampicillin, tetracycline, compound trimethoprim/sulfamethoxazole, and amoxicillin.

Based on statistical analysis, the following correlations were identified: (a) expression of the *fim*C gene and resistance to ciprofloxacin (*p = *.001), gentamicin (*p = *.001), amikacin (*p = *.001), levofloxacin (*p = *.001), and streptomycin (*p = *.001); (b) expression of the *fim*A gene and resistance to tetracycline (*p = *.001), ampicillin (*p = *.001), compound trimethoprim/sulfamethoxazole (*p = *.001), and amoxicillin (*p = *.003); (c) expression of the *agg* gene and resistance to gentamicin (*p = *.001), tetracycline (*p = *.001), ciprofloxacin (*p = *.017), and levofloxacin (*p = *.001); and (d) expression of the *stx*2 gene and resistance to ampicillin (*p = *.001), tetracycline (*p = *.001), compound trimethoprim/sulfamethoxazole (*p = *.002), and amoxicillin (*p = *.015; Table 10).

**Table 10:** Distribution of antimicrobial resistance among virulence factor^a^

Antibiotic	AMP	TET	STR	GEN	CIP	LEV	AMK	T/S	AMX
*fim *C (*n* = 52)									
Positive, %	23 (44.2)	25 (48.1)	12 (23.1)^b^	1 (1.9)^b^	6(11.5)^b^	5 (9.6)^b^	2 (3.8)^b^	18(34.6)	16 (30.8)
*p* Value	.592	.352	.001	.001	.001	.001	.001	.224	.056
*fim *A (*n* = 19)									
Positive, %	6 (31.6)^b^	7 (36.8)^b^	1 (5.2)	1 (5.3)	2 (10.5)	2 (10.5)	3(15.8)	7 (36.8)^b^	4 (21)^b^
*p* Value	.001	.001	.307	.165	1.000	.241	.107	.001	.003
*agg *(*n* = 34)									
Positive, %	11 (32.4)	15 (44.1)^b^	7 (20.6)	1 (2.9)^b^	3(8.8)^b^	5(14.7)^b^	0 (0)	10 (29.4)	7 (20.6)
*p *Value	.051	.001	.169	.001	.017	.001	/	.204	.566
*stx*2 (*n* = 21)									
Positive, %	8 (38.1)^b^	7 (33.3)^b^	4 (19)	1 (4.8)	0 (0)	1(4.8)	0 (0)	4(19)^b^	4 (19)^b^
*p* Value	.001	.001	.619	.057	/	.091	/	.002	.015

Abbreviations: AMK, amikacin; AMP, ampicillin; AMX, amoxicillin; CIP, ciprofloxacin; GEN, gentamicin; LEV, levofloxacin; STR, streptomycin; T/S, cotrimoxazole; TET, tetracycline.

^a^ Data are presented as No. (%).

^b^ Statistically significant.

## 4 CONCLUSIONS

Differences in the pathogenicity of *E. coli* and its susceptibility to antimicrobial agents were detected in different retail foods. This must be taken into account in developing guidelines for retail food management. Periodic review and formulation of antibiotic consumption policies are required to control the spread and acquisition of antibiotic resistance. Because most isolates express several types of VFs at the same time, it is necessary to further study the interaction between different VFs at the molecular level.

In conclusion, *E. coli* has become a potential source of foodborne illness due to the possibility of horizontal transfer of drug‐resistant genes, high drug resistance rate, and the correlation between the resistance to some antibiotics and several virulence factors. As those problems become more and more serious, we need to strengthen the supervision of veterinary drugs used in the raising of livestock. At the same time, the detection and monitoring of antimicrobial agents in animal foods can help to reveal the ongoing use of prohibited animal husbandry practices.

## CONFLICT OF INTEREST

The authors declare that they do not have any conflicts of interest.

## ETHICAL STATEMENTS

This study did not involve any human or animal testing.
